# Multiple Correspondence Analysis on Amino Acid Properties within the Variable Region of the Capsid Protein Shows Differences between Classical and Virulent Systemic Feline Calicivirus Strains

**DOI:** 10.3390/v11121090

**Published:** 2019-11-23

**Authors:** Sylvie Brunet, Cécile Sigoillot-Claude, Daniel Pialot, Hervé Poulet

**Affiliations:** Boehringher Ingelheim, Animal Health, 69800 Lyon, France; cecile.sigoillot-claude@boehringer-ingelheim.com (C.S.-C.); daniel.pialot@orange.fr (D.P.); herve.poulet@boehringer-ingelheim.com (H.P.)

**Keywords:** Feline Calicivirus, pathotypes, viral systemic disease, phylogeny, multivariate statistical analysis, JAM-1, VP1, sequence

## Abstract

Feline calicivirus (FCV) is a widespread and highly prevalent pathogen of domestic cats, responsible for mild upper respiratory tract disease. Outbreaks of severe virulent systemic disease (VSD) associated with FCV infection have been reported worldwide. VSD FCV strains have a broader tropism and cause a systemic vascular compromise. Despite clear differences in the pathogenesis of VSD and oral respiratory infections, attempts to identify specific molecular markers of VSD strains on the major capsid protein VP1 have failed. Region E of VP1 is responsible for the interaction with the cell receptor Junctional Adhesion Molecule JAM-1 (FeJAM-1) and with VP2 minor capsid protein during the entry of the virus. We carried out an original analysis on the sequences from region E of VSD and classical strains. A Multiple Correspondence Analysis was performed on a Boolean matrix built by coding sequences on the basis of their amino acid properties. For the first time, this approach was able to differentiate VSD and classical FCV. Seven remarkable residue positions were shown to be statistically significant for pathotype differentiation, mainly located in the N-terminal hypervariable part of region E. As structural analysis suggested an interaction of these residues with FeJAM-1 or VP2, post-binding events, and specific conformational changes may explain the difference of pathogenesis between pathotypes.

## 1. Introduction

Feline Calicivirus (FCV) is a small RNA non-enveloped virus belonging to the Caliciviridae family. The single strand RNA genome contains three open reading frames (ORF) coding for non-structural proteins (ORF1), VP1 major capsid protein (ORF2), and VP2 minor capsid protein (ORF3) respectively. The *T* = 3 icosahedral capsid is made of 90 dimers of VP1 [1]. VP2 is also incorporated into the virion at a lower copy-number [2]. FCV displays a remarkable genetic and antigenic diversity [3,4,5,6]. The region E encompassing amino acids 426 to 521 in the protruding domain of VP1 [7,8] is the major contributor to this variability. Two hypervariable domains have been described in this region E, namely *N*-HV (aa 426–460) and *C*-HV (aa 490–521), separated by a short conserved area. The hypervariable domains contain the immunodominant neutralizing epitopes of the virus [9,10]. Region E is also involved in the recognition and interaction with the cell receptor, feline Junctional Adhesion Molecule JAM-1 (FeJAM-1), for virus entry by endocytosis [11,12,13]. FeJAM-1 is a transmembrane glycoprotein, involved in tight junctions of epithelial and endothelial cells. FCV binds also to α-2,6-sialic acid [14].

FCV is a widespread and highly prevalent pathogen of domestic cats, responsible for mild upper respiratory tract disease characterized by oral ulcers, nasal discharge, and conjunctivitis [15,16]. FCV infection can also result in chronic stomatitis [17,18,19]. Many cats are asymptomatic carriers contributing to the diffusion of the virus within the cat population [20,21]. Since 1998, outbreaks of severe virulent systemic disease (VSD) associated with FCV infection have been reported in the USA and then in Europe [22,23,24,25,26,27,28,29]. VSD FCV strains have a broader tropism and infect both epithelial and endothelial cells causing a systemic vascular compromise [22,30]. As a consequence, the infected cats suffered from facial and limb edemas, high fever, icterus, pancreatitis, and hemorrhages. In contrast with classical FCV-induced oral respiratory disease, VSD comes with a high mortality rate, up to 60%.

Despite clear differences in the pathogenesis of VSD and oral respiratory infections, attempts to identify specific molecular or genetic markers of VSD strains on the major capsid protein VP1 or related ORF2 gene have failed. Phylogenic analyses of ORF2 gene or its region E have confirmed the strong variability of FCV whichever the associated pathology but have not been able to identify a VSD strains cluster [26,31,32]. Although some amino acid substitutions have been reported in the N-terminal hypervariable area of region E of some VSD strains [26,28], they were not consistently observed in other VSD strains.

Since the region E of VP1 is responsible for the interaction with FeJAM-1 receptor and with VP2 during the entry of the virus into the cell [33], we speculated that this region harbors different properties between VSD and classical strains. As standard phylogenic analyses failed to identify those potential differences, we carried out an original analysis of the protein sequences of the region E of VSD and classical strains based upon the properties of the amino acids instead of their identity only. The sequences were coded for amino acid properties into a Boolean matrix then compared by a statistical Multiple Correspondence Analysis (MCA), to sort sequences depending on the amino acid characteristics and to determine statistically significant positions.

## 2. Materials and Methods

### 2.1. Sequences

Fifty seven sequences of the hypervariable region E were included in the analysis: 16 sequences of VSD FCV and 41 sequences of FCV classical oral respiratory disease (referred as ORD FCV), either from published sequences or from sequencing after viral isolation. All strains were isolated between 1998 and 2015 from several countries worldwide. The MLV vaccine strain F9 was also included. The length of the region E was 94 to 98 amino acids (aa), spanning positions 426 to 521 of the major capsid protein precursor (VP1-668 amino acids). When available, 31 sequences of the full VP1 capsid protein were also submitted to phylogenic analysis.

The characteristics and access number of the sequences are summarized in Table 1.

### 2.2. Virus Collection

FCV strains were isolated from cats suffering from either ORD (strains Kit426, Izzy) or VSD (strains Mimi, Felix, 2359, OBI, EN, Bretagne) from France or USA, between 2001 and 2014 (Table 1). Strains 100869, 88287, 33585 (VSD strains, USA, 2001) were obtained from Cornell University.

### 2.3. Virus Isolation

FCV strains were isolated from field cats suffering from ORD or VSD. The tissues (kidney, skin biopsy) were grinded in culture medium F15 supplemented with antibiotics and 5% fetal calf serum and in the presence of a cat serum specifically neutralizing feline herpes virus. Oropharyngeal swabs were swollen in the same medium. After centrifugation, supernatant was inoculated onto a monolayer of CRFK (Crandell Reese Feline Kidney) cells in T25 flasks. After incubation at 37 °C for 1 to 4 days, the supernatant from inoculated cells was collected when a typical FCV cytopathic effect (CPE) reached 80% of the cells layer. A second passage on cells was performed in the same conditions.

### 2.4. Sequencing of Region E

A fraction of ORF2 including region E was amplified by RT-PCR after extraction of viral RNA from FCV isolates, using the extraction kit QiAmp Viral RNA (Qiagen, Courtaboeuf, France) according to the manufacturer instructions. The RT-PCR was performed on 5 μL of viral RNA in a total volume of 25 μL with the primer set F1244 5′-GATCCCTGATGGTTGGCC-3′ (forward)/R1949 5′-ATTCCCATGTAGGAGGC-3′ (reverse) or with the primer set F832 5′-CAYCTDATGTCTGAYACTG-3′ (forward)/R1963 5′-GAATTCCCATGTAGGAGGC-3′ (reverse), using the kit PHUSION RT-PCR (Thermo Fisher Scientific, Waltham, MA, USA) according to the manufacturer instructions (proofreading Taq polymerase). The resulting 706 bp (first set of primers) or 1131 bp (second set of primers) amplification band was visualized on agarose electrophoresis with ethidium bromide straining. The RT-PCR products were directly sequenced on both strands, using the same primers (Eurofins Genomics, Anzinger, Germany). The sequences were assembled and translated (software Vector NTI, Thermo Fisher Scientific, Waltham, MA, USA) to obtain the protein sequence of the region E part of VP1.

### 2.5. Phylogenic Analysis

The sequences of the full VP1 capsid protein or restricted to its variable region E of the strains isolated in this study were compared with published strains, from several countries and isolated in the 2000s (as described in Table 1). The sequences were aligned by ClustalW2 program (Software Seaview, version 4.3.5 [34]) and their percentages of identity were calculated. A dendrogram was built, based upon Neighbor-Joining distance method with Kimura correction, using 500 replicates for bootstrap analysis (Seaview). Bootstrap values were considered significant when superior to 70. For analysis of the full VP1 capsid protein sequences, the tree was rooted with a Canine Vesivirus major capsid protein sequence, whereas the region E-tree remained unrooted as sequences were too divergent on this part of the protein to be computed with an external root.

### 2.6. Multiple Correspondence Analysis (MCA) of Region E

The alignment of all region E sequences was converted into a matrix for MCA analysis [35]. First, a 57 × 98 table was built from aligned sequences: each raw was a strain sequence (57 raws), each column was an amino acid (98 columns). Each amino acid can be described by a set of nine properties, i.e., hydrophobic, positive, negative, polar, charged, small, aromatic, aliphatic, proline, as shown in Table 2. The initial amino acids table was then converted into a 57 × 882 Boolean matrix based on amino acid properties, replacing each amino acid column for each alignment position by nine columns corresponding to the nine properties, coded 0 or 1 if property was false or true respectively.

The MCA was performed on these data using R Stats software (R Stats 3.6.1 —r.project.org; software JMP—S.A.S institute JMP). The objective of this multivariate analysis applied to categorical variables was to identify the multiple correspondences between the levels of the categorical variables. The correlation ratios between the MCA components and the categorical variables were used to identify the variables (amino acid position and properties) which were more likely to be correlated to the VSD or ORD pathotype. The correlations measured by MCA were confirmed with contingency tables between each variable and the “pathotype” variable. Fisher exact test was performed to determine which variables were significantly linked to the “pathotype” variable. The *p*-values were corrected by Benjamini–Hochberg (BH) method to keep the level of false alarms close to 5% when a large number of tests are performed. The structural correlations between the significant categorical variables were confirmed by contingency tables and chi-square significance tests. The MCA analysis was performed by Ippon Innovation Company (Toulouse, France).

### 2.7. Structural Impact of Mutations

Two different structures published in the Protein Data Bank (PDB) were used as template to model the different structures of the FCV variants: 6GSH structure for the pre-fusion form of VP1 and 6GSI for the post-fusion form of VP1 and VP2 after decoration of the virion with the soluble ectodomain of FeJAM-1 [33]. Modeling of VP1 structure was conducted upon these templates with Swiss-model automated protein structure homology-modelling server [36]. Models were visualized using Maestro 11.8 software and sur-impressed in order to localize the amino acids highlighted by MCA analysis and identify their chemical interactions with other residues in the pre fusion and post fusion states.

## 3. Results

### 3.1. Phylogeny

**Figure 1:**
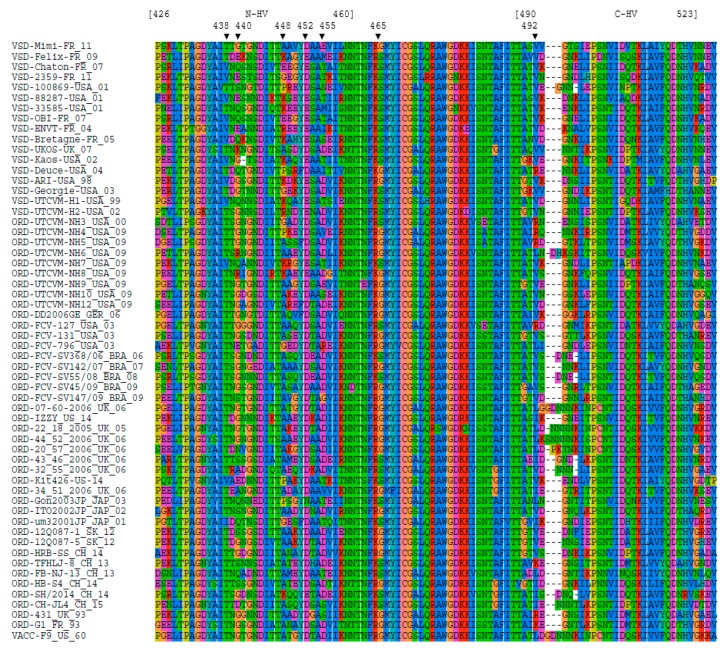
Alignment of region E. The sequences are recorded as “pathotype (oral respiratory disease (ORD) or virulent systemic disease (VSD))”-“strain”_”country of isolation”_”year of isolation”. The two hypervariable (“HV”) parts of the region E are identified (*N*-HV and *C*-HV), positions referring to the capsid protein VP1 precursor (ORF2). The seven positions shown to be significant from the Multiple Correspondence Analysis (MCA) analysis are indicated by vertical arrows.

The alignment of region E of the 57 FCV strains is shown in Figure 1. As expected, region E of the capsid protein is highly variable especially within domains 426–460 (*N*-HV part) and 490–523 (*C*-HV part), which are separated by a less variable region (aa 461–489). Some VSD strains showed characteristic amino acids (438 T, 448 A, 465 S) but these positions were not full VSD markers as not found for all VSD strains and also sometimes found on ORD strains.

Overall, VP1 sequence identity was 86.1 ± 2%. In region E, sequence identity is reduced to 69.6 ± 5 % (56–98%). Variability within region E was similar whichever the pathotype of the strain (70.9 ± 4% within VSD strains, 70.1 ± 5% within ORD strains, 68.7 ± 4% between VSD and ORD strains). The same observation could be done with the full VP1 protein (87.6 ± 2% within VSD strains, 85.6 ± 2% within ORD strains, 86.3 ± 2% between VSD and ORD strains).

**Figure 2:**
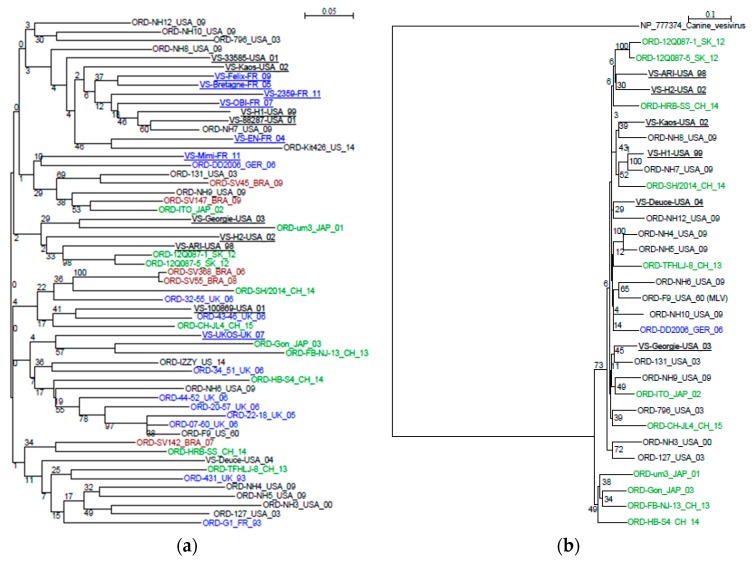
Phylogenic trees for Capsid Protein and region E. (**a**) region E; (**b**) capsid protein; bold and underlined: VSD strains; black: US strains; blue: EU strains; green: Asia strains; red: Brazil strains. Bootstrap values superior to 70 are considered significant. The nomenclature for the sequences is the same as reported in Figure 1.

The dendrograms for either VP1 protein or for region E are shown in Figure 2. The phylogenic analysis did not identify a cluster of VSD strains. For instance, the VP1 tree showed subgroups combining both VSD and ORD strains: VSD Deuce with ORD NH12, VSD Georgie with ORD 131, VSD Kaos with ORD NH8. VSD Ari and VSD H2 were the only VSD which sub-clustered together. In the phylogenic tree based on region E sequences, no cluster could be identified and bootstrap values remained very low, as the result of the high variability in a fairly short domain.

### 3.2. MCA Analysis on Amino Acids Properties

**Figure 3:**
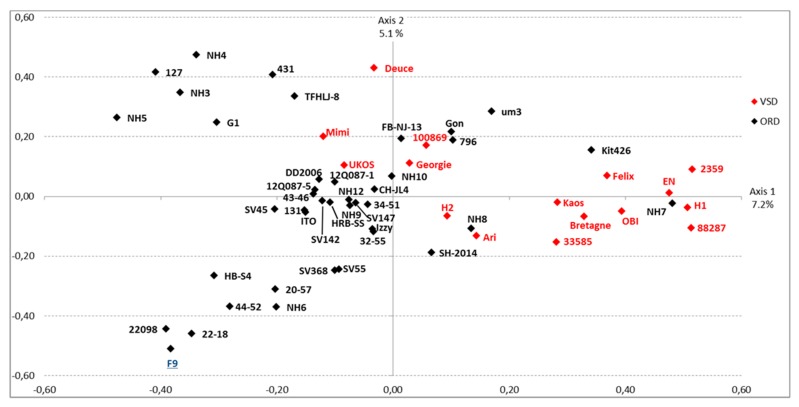
MCA graph. The graph presents the sequences sorted after MCA analysis on the basis of the residue properties of region E domain. The horizontal axis is able to differentiate ORD strains (black) and VSD strains (red). The vaccine strain F9 (attenuated ORD strain) is shown in blue.

A first MCA analysis was performed on amino acid properties of the compared sequences, considering these properties as categorical variables, independently of the associated pathotype. The resulting graph is shown in Figure 3. The expressed percent of variance is 7.2% for the first axis and 5.1% for the second axis. These values are low as the MCA was applied to a high number of categorical data (882 variables). On the first axis, MCA analysis was able to differentiate VSD strains and ORD strains, even in a blind analysis (as the pathotype was not included in the set of variables). The abscise values were submitted to an ANOVA (analysis of variance): the mean value for abscises of ORD sequences was significantly different from the mean value for abscises of VSD sequences (*F*-test, *p* < 0.0001), confirming the segregation of ORD and VSD sequences.

However, a few sequences remained undifferentiated, located near the center of the graph (VSD strains: Mimi, Ukos, Deuce and for ORD strains: Kit426, NH7, Gon, um3, 796, NH8, SH-2014).

A second MCA was conducted by adding the “pathotype” as an additional variable to the amino acid properties variables. The results confirmed the first analysis. The consistency of both analyses highlighted the fact that the amino acid properties were valuable factors for discriminating between the two pathotypes.

A combination of 13 amino acid properties was found to be significantly associated with the pathotype of the strain, corresponding to seven remarkable positions in the capsid protein (Table 3 and Figure 1): amino acid positions 438, 440, 448, 452, and 455 in the *N*-HV part of region E, 465 in the central conserved part, 492 in the *C*-HV part of region E. Two complementary MCA were performed on *N*-HV domain only and then on *C*-HV domain only. The significant contributors were the same as in the MCA on the entire region E, thus confirming the highest contribution of the *N*-HV part. The properties of the 13 identified amino acids are described in Table 3.

According to the MCA, VSD strains are more likely to have a combination of the following properties: non polar amino acid with aliphatic chain in position 438, not a small amino acid in position 440, a polar charged positive amino acid in position 448, not a small amino acid at position 452, not a charged negative amino acid at position 455, a polar amino acid at position 465 and a small amino acid at position 492.

**Figure 4:**
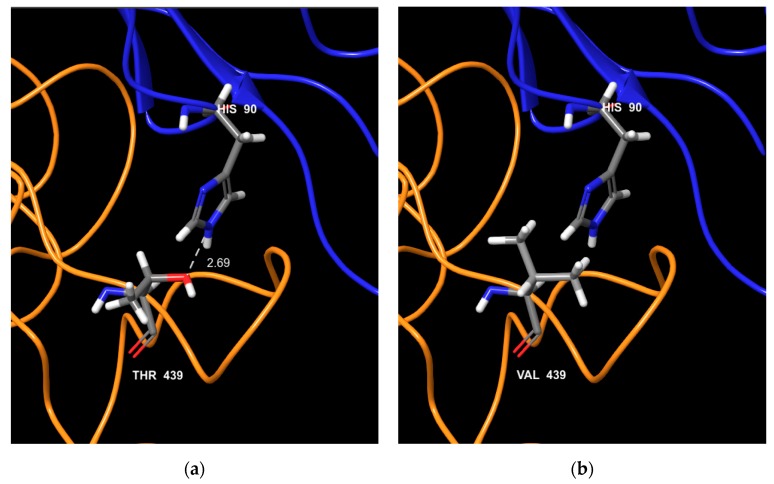
Interaction between Feline calicivirus (FCV) VP1 and Jam-1 receptor for ORD and VSD strains. FCV VP1 (orange chain) aa 438 (labelled 439 in the figure as a one amino acid shift is present in the 6GSI sequence compared to other strains used in this article) and Jam-1 receptor (blue chain) for ORD FCV typical strains (**a**) and VSD typical strain (**b**). For the ORD strain, both aa have redox properties and could create an electric bond. For the VSD strain, as the aa 438 does not have redox properties, no bond could be created. Amino acid color code: grey bar for carbon; blue bar for nitrogen; red bar for oxygen; white bar for hydrogen.

**Figure 5:**
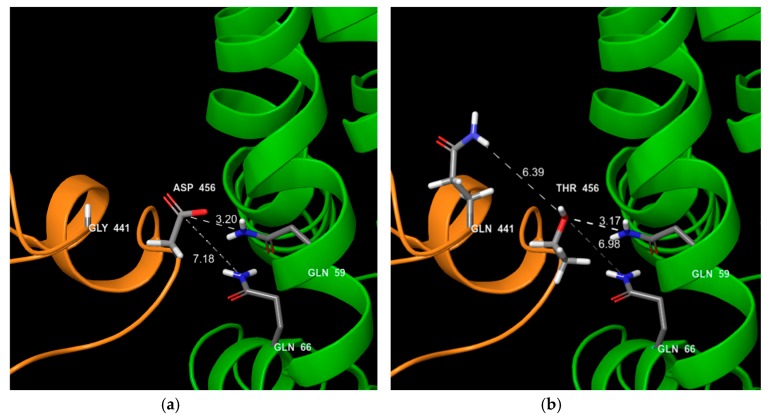
VP1 interactions with VP2 ORD FCV typical strain and VSD typical strain. Orange chain: VP1; green chain: VP2; (**a**) ORD strain; (**b**) VSD strain. For ORD strains, aa440 (441 in the 6GHI sequence) is generally a glycine and for VSD a glutamic acid or a glutamine. The aa455 (456 in the 6GHI sequence) is generally an aspartic acid for ORD strains and generally a threonine for VSD. For the ORD strain, no interaction is shown between VP1 gly440 and thr456. In contrast, for VSD, an interaction could occur between gln441 and thr455 and may subsequently consolidate the interaction between VP1 and VP2.

Residue 438 is a key element for the interaction between VP1 and the FeJAM-1 receptor. According to 6GSI structure [33], residue 438 of VP1 interacts with His90 of FeJAM-1 with less than 4 angstroms distance between the two amino acids. In ORD strains, residue 438 is usually a Threonine (38 ORD strains out of 41), a polar amino acid, H^+^ donor or acceptor. In VSD strains, Thr438 was generally mutated into Val438 an aliphatic hydrophobic amino acid with no redox properties (nine VSD strains out of 17). When these variations are reported on the structure (Figure 4), the loss of electronic bound in the VSD strains could affect the interaction with the receptor. Based on post fusion structure 6GSI, the amino acid residue 455 should interact with two VP2 residues (Figure 5): Gln59 and Gln66, both H^+^ donors. In VSD strains, this Asp455 is mainly mutated into Thr455 or Ser455 (seven VSD strains). Threonine and Serine have H^+^ donor and acceptor properties and an hydroxyl component, which could enhance the binding properties to a H^+^ donor and acceptor. In VSD strains, intra-chain interaction could occur between VP1 Gln440 and Thr455, decreasing the flexibility of the loop whereas Asp455 from ORD strains has no interaction with Gly440 (Figure 5). Amino acid residues 448 and 465 have no direct interaction with FeJAM-1 nor VP2 but these residues are located on the protein surface. For ORD strains, residue 448 is frequently an alanine (32 ORD/41), a neutral amino acid while it is usually a polar positively charged amino acid in VSD FCV strains (9 VSD/17, for Arg and Lys). Amino acid 465 is generally a Glycine in ORD strains (27 ORD/41) and a serine acting as an H^+^ acceptor/donor in VSD FCV strains (14 VSD/17). These two mutations in the same surface area increase the possibility to establish hydrogen bonds with other residues.

## 4. Discussion

FCV-induced VSD was first reported in 2000 [22] and outbreaks or foci of VSD have then been regularly reported in different regions of the world (i.e., North America, Europe, Japan, China, etc.) [22,26,29,30,31,32]. VSD FCV strains have a different phenotype than ORD, with a more efficient replication in cell culture in vitro [37]. Accordingly, viremia after experimental infection was shown to be higher in VSD FCV infected cats than in classical strains [38]. Viral loads in infected organs are usually high [28] and significantly more virus was excreted following experimental infection with VSD FCV compared to classical strains of FCV [28,39].

Another characteristic of VSD FCV strains is their broader tropism. The virus has been found in both endothelial and epithelial cells and can infect multiple organs [22,27,28,29,30,40,41].

Since the emergence of VSD strains, all attempts to identify genetic markers specific of hypervirulent strains within the capsid protein have been unsuccessful [25,26,27,28,29,31,32,41,42,43,44]. Previous attempts to find some correlation between distinct disease manifestations and genetic traits had also failed [45]. In this study, we confirmed that classical sequence analyses based on VP1 or its region E do not allow to cluster the FCV VSD pathotype. A few mutations have been reported in the VP1 of some VSD FCV strains. For example, FCV-Ari and FCV-Kaos share similar amino acids substitutions at positions 398, 430, 438, 448, 452, 581, and 592 [26]. However, these changes were not found in other FCV VSD strains and some of them could be observed in classical strains [29,32,41].

To address this chaotic picture, we used a multiple correspondence analysis (MCA) based on the amino acid physico-chemical properties of region E of VP1. The analysis was focused on region E (426–523) which contains the two hypervariable domains responsible for most of the antigenic variability and is involved in the binding to the FeJAM-1 receptor. The analysis allowed the identification of a set of amino acid positions for which some physico-chemical properties are more frequently observed in VSD strains. No amino acid position could be considered as a reliable isolated marker by itself. For example, V438 is frequently observed in VSD strains but it can occasionally be found in ORD strains and is missing in some VSD strains (Figure 1).

Most positions are located in the hypervariable domains of region E, mainly in the *N*-HV domain except for one in the *C*-HV domain and one in the more conserved central area. Region E is exposed at the surface of the distal P2 subdomain of VP1. It is involved in the interaction with FeJAM-1 receptor [13,46]. A set of interacting amino acids within the P2 subdomain of VP1 and D1 Ig-like domain of FeJAM-1 have been predicted by cryo-electron microscopy reconstructions and structural fitting experiments [46]. Docking experiments suggested that most contact residues were located in the N-terminal hypervariable domain of region E. Interestingly, most of the positions identified by the MCA are located in the same domain, suggesting possible differences in the way VSD and ORD FCV interact with FeJAM-1. In the MCA, residue 438 of region E has been identified within the set of amino acid positions contributing to the discrimination between VSD and ORD FCV strains. This residue is expected to interact with His90 of FeJAM-1. This residue belongs to the loop of P2 subdomain, implied in structural changes upon receptor binding [1]. Transfection experiments in different cell lines with feJAM-1 highlighted differences of infectivity between FCV isolates but failed to distinguish between VSD and ORD FCV strains in their ability to infect the transfected cells [12]. All isolates were able to infect at least one of the FeJAM-1 expressing cell lines. Therefore, binding sensus-stricto to FeJAM-1 is probably not a distinctive feature of VSD FCV strains.

Soluble Fe-JAM-1 can neutralize VSD strains in vitro at 37 °C but not ORD strains [37]. Mutations of residues exposed on the surface of P2 could make the VSD FCV-5 strain resistant to neutralization by soluble FeJAM-1. Some of those mutations concern residues identified in the MCA (T438I) or in their vicinity (N443S). The binding of VP1 to FeJAM-1 induces conformational change of VP1, with a rotation of its protruding domain (loop 436–448 of P2) and gain of flexibility [33,37]. The interaction between FCV and soluble FeJAM-1 triggers changes in the surface hydrophobicity of VP1 that depend on the strain. Preliminary experiments suggest that this increase of surface hydrophobicity is stronger in VSD FCV than in ORD FCV strains [37]. By mutating residues predicted to interact with FeJAM-1 or residues in the conserved domain of region E of a FCV-5-Urbana chimera, Lu and colleagues identified amino acid positions which would play a role in the post-binding events [13]. Two of them were located close to positions identified in the MCA (I437A; K493E). Post-binding events may explain the difference of pathogenesis between VSD and ORD FCV. RNA release assays showed that, on the contrary to ORD FCV strains, VSD FCV strains do not require a low pH to release their genomic RNA, suggesting that conformational changes in the capsid after binding to FeJAM-1 are different between the two pathotypes [47].

The VP2 minor capsid protein is critical for the production of infectious viral progeny [2]. Cryo-electron microscopy studies showed that VP2 forms a portal-like assembly following the interaction between VP1 and its FeJAM-1 receptor [33]. Three amino acids identified in the MCA interact with VP2 protein (residues 440, 452, and 455 of the *N*-HV part of region E). Further studies will be necessary to understand the impact of these mutations on the interaction between VP1 and VP2. We may speculate that the physico-chemical properties of these amino acids are critical for the stabilization of the VP1-VP2 post-fusion complex and would allow the portal entry to be assembled at neutral pH. As a result, VSD FCV strains’ infectivity would be enhanced, consistently with their phenotype. This would also explain the ability of soluble FeJAM-1 to neutralize VSD FCV strains, as a result of profound alteration of the capsid proteins after binding to the receptor.

The MCA was able to differentiate VSD and ORD sequences. It is the first time a statistical method based on amino acids sequence of region E is able to discriminate VSD and ORD FCV isolates. Nevertheless, some VSD or ORD strains could not be clustered within their respective group. A possible explanation may be related to the absence of a strictly univocal definition of the virulent systemic disease. The main difference with ORD is the involvement of other tissues or organs than the upper-respiratory tract. VSD is a combination of clinical signs like fever, oral ulcers, facial and/or limb edema, skin ulcerations, and clinical signs associated with multiple organ dysfunction (i.e., lungs, liver, pancreas, etc.). However, all clinical signs are not systematically observed, as illustrated in some outbreaks [41]. Along with these clinical differences, the virulence of VSD FCV strains is variable. For instance, the FCV-100869 VSD strain can induce VSD when administered at high titer (10^8^ CCID_50_) but induces a classical ORD at lower titers, while FCV-33585 induces VSD even at lower titers (10^5^ CCID_50_) [38]. On the other hand, strains reported to be isolated from a cat with upper-respiratory tract disease may be found to be hypervirulent when inoculated to cats, like strain 393 used in a vaccine efficacy study [48]. Another possible cause of unsuccessful clustering might be mutations of the isolate during passages in cell culture before sequencing.

## 5. Conclusions

This is the first study describing a method to discriminate between VSD and ORD FCV isolates. It confirms that there is no evident genetic marker of the VSD pathotype. On the contrary, the difference between classical and hypervirulent FCV strains lies in physico-chemical properties of a combination of amino acids within region E of VP1. These amino acids belong to a region which is involved in the interaction with FeJAM-1 and/or VP2 during virus entry into the cell. These mutations may explain the differences of viral fitness and infectivity. However, they do not explain the difference of tropism between VSD and ORD strains. Further work will be needed to find out why ORD FCV strains which can use FeJAM-1 have a tropism more or less restricted to the upper-respiratory tract. This work may however be complicated by the fact that there is not a clear and strict distinction between VSD and ORD FCV strains, both at the clinical and at the molecular level.

## Figures and Tables

**Table 1:** Characteristics of the sequences.

Strain	Pathotype	Country	Year	Access Number	Length (aa)	Strain	Pathotype	Country	Year	Access Number	Length (aa)
Mimi	VSD	France	2011	MN628612	95	SV368	ORD	Brazil	2006	AEO16222	95
Felix	VSD	France	2009	MN628608	95	SV142	ORD	Brazil	2007	AEO16224	95
2359	VSD	France	2011	MN628602	95	SV55	ORD	Brazil	2008	AEO16226	95
100869	VSD	USA	2001	MN628605	95	SV45	ORD	Brazil	2009	AEO16230	95
88287	VSD	USA	2001	MN628604	95	SV147	ORD	Brazil	2009	AEO16231	95
33585	VSD	USA	2001	MN628603	95	07-60	ORD	UK	2006	AFR44751	98
OBI	VSD	France	2007	MN628613	95	IZZY	ORD	USA	2014	MN628610	95
EN	VSD	France	2004	MN628607	95	22-18	ORD	UK	2005	AFR44753	97
Bretagne	VSD	France	2005	MN628606	95	44-52	ORD	UK	2006	AFR44754	98
UKOS	VSD	UK	2007	ACV81957	95	20-57	ORD	UK	2006	AFR44756	97
Kaos	VSD	USA	2002	ABI84214	94	43-46	ORD	UK	2006	AFR44762	95
Deuce	VSD	USA	2004	ABI84202	95	32-55	ORD	UK	2006	AFR44768	95
ARI	VSD	USA	1998	ABI84212	95	Kit426	ORD	USA	2014	MN628611	95
Georgie	VSD	USA	2003	ABI84206	95	34-51	ORD	UK	2006	AFR44771	95
H1	VSD	USA	1999	AAT66087	95	Gon	ORD	Japan	2003	AHZ59401	95
H2	VSD	USA	2002	AAT66090	95	ITO	ORD	Japan	2002	AHZ59402	95
NH3	ORD	USA	2000	AAT66084	95	um3	ORD	Japan	2001	AHZ59403	95
NH4	ORD	USA	2009	AAY44304	95	12Q087-1	ORD	South-Korea	2012	AIA09956	95
NH5	ORD	USA	2009	AAY44305	95	12Q087-5	ORD	South-Korea	2012	AIA09959	95
NH6	ORD	USA	2009	AAY44306	97	HRB-SS	ORD	China	2014	AII00833	95
NH7	ORD	USA	2009	AAY44307	95	TFHLJ-8	ORD	China	2013	AIN37114	95
NH8	ORD	USA	2009	AAY44308	95	FB-NJ-13	ORD	China	2013	AIS22460	95
NH9	ORD	USA	2009	AAY44309	95	HB-S4	ORD	China	2014	ALI87297	95
NH10	ORD	USA	2009	AAY44310	95	SH-2014	ORD	China	2014	ALM55428	95
NH12	ORD	USA	2009	AAY44312	95	CH-JL4	ORD	China	2015	ALO69832	96
DD2006	ORD	Germany	2006	ABD84433	95	G1	ORD	France	1993	MN628609 MN628601	95
127	ORD	USA	2004	ABI84196	95	431	ORD	UK	1993		95
131	ORD	USA	2004	ABI84198	95	F9	Vaccine	USA	1960	CAA77636	98
796	ORD	USA	2003	ABI84200	95						

Access number for protein sequence; underlined strains: full capsid protein sequence is available (same access number). Disease: VSD = Viral Systemic Disease; ORD = Oral Respiratory Disease.

**Table 2:** Coding table for amino acid properties.

Amino Acid	aa Properties									
	Hydrophobic	Positive	Negative	Polar	Charged	Small	Aromatic	Aliphatic	Proline	Deletion
A	1	0	0	0	0	1	0	0	0	0
C (*)	1	0	0	0	0	1	0	0	0	0
D	0	0	1	1	1	1	0	0	0	0
E	0	0	1	1	1	0	0	0	0	0
F	1	0	0	0	0	0	1	0	0	0
G	1	0	0	0	0	1	0	0	0	0
H	1	1	0	1	1	0	1	0	0	0
K	1	1	0	1	1	0	0	0	0	0
I	1	0	0	0	0	0	0	1	0	0
L	1	0	0	0	0	0	0	1	0	0
M	1	0	0	0	0	0	0	0	0	0
N	0	0	0	1	0	1	0	0	0	0
P	0	0	0	0	0	1	0	0	1	0
Q	0	0	0	1	0	0	0	0	0	0
R	0	1	0	1	1	0	0	0	0	0
S	0	0	0	1	0	1	0	0	0	0
T	1	0	0	1	0	1	0	0	0	0
V	1	0	0	0	0	1	0	1	0	0
W	1	0	0	1	0	0	1	0	0	0
Y	1	0	0	1	0	0	1	0	0	0

(*) C as a cystine form.

**Table 3:** Correlation table.

Variable	VSD Strain	*p*-Value
448_POLAR	Y	<0.001
448_CHARGED	Y	<0.001
448_SMALL	N	<0.001
452_SMALL	N	<0.001
448_POSITIVE	Y	0.005
438_POLAR	N	0.014
438_ALIPHATIC	Y	0.014
492_SMALL	Y	0.014
465_HYDROPHOBIC	N	0.025
465_POLAR	Y	0.025
440_SMALL	N	0.027
455_CHARGED	N	0.034
455_NEGATIVE	N	0.043

The variables are recorded as amino acid position on capsid protein and associated property. The *p*-values were corrected by Benjamini–Hochberg method. For each of the properties, the preferred profile of the VSD strains is indicated (Y = yes; N = no).
